# Drug-induced pulmonary edema: a real-world pharmacovigilance study using the FDA Adverse Event Reporting System (FAERS)

**DOI:** 10.1590/1414-431X2025e14566

**Published:** 2025-08-22

**Authors:** Zilan Zhong, Manting Liu, Qian Zhong, Miao Zhou, Xingwei Di

**Affiliations:** 1 Shenzhen Clinical Medical College, Guangzhou University of Chinese Medicine, Shenzhen, China; 2 Clifford Hospital, Guangzhou University of Chinese Medicine, Guangzhou, China; 3 The First Affiliated Hospital of Jinzhou Medical University, Jinzhou, China; 4 Foshan Hospital of Traditional Chinese Medicine, Foshan, China

**Keywords:** Pulmonary edema, Adverse drug reactions, Pharmacovigilance, Disproportionality analysis

## Abstract

This study aimed to systematically evaluate the risk of drug-induced pulmonary edema (DIPE) using the Food and Drug Administration (FDA) Adverse Event Reporting System (FAERS) database. This retrospective pharmacovigilance study utilized FAERS data from the first quarter of 2004 to the second quarter of 2024. We identified drugs with at least 10 reported DIPE cases as primary suspects (PS). The DIPE signals were assessed using four methods: Reporting Odds Ratio (ROR), Proportional Reporting Ratio (PRR), Bayesian Confidence Propagation Neural Network (BCPNN), and Empirical Bayes Geometric Mean (EBGM). Multivariate logistic regression was employed to control for confounding factors, and the timing of DIPE onset was statistically analyzed. Out of 173 target drugs, 37 were identified with DIPE risk. The top five drugs were naloxone, dasatinib, nifedipine, anti-thymocyte globulin, and pioglitazone. Multivariate logistic regression indicated that all except pioglitazone were independent risk factors for DIPE. The onset time of DIPE varied by age and gender for some drugs. This study is the first to identify the DIPE risk systematically associated with multiple drugs. It highlights the need for clinicians and pharmacists to be aware of these high-risk drugs and to monitor high-risk populations closely to ensure medication safety.

## Introduction

Non-cardiogenic pulmonary edema (NCPE) is a prominent manifestation of drug-induced lung injury, characterized by dyspnea, chest discomfort, tachypnea, and hypoxemia. Radiographic findings typically show interstitial and alveolar infiltrates. Unlike cardiogenic pulmonary edema (CCPE), which results from heart failure and elevated pulmonary pressures, NCPE arises from disruption of the alveolar-capillary barrier, leading to fluid leakage into the alveolar spaces without the involvement of cardiac dysfunction. Drug-induced pulmonary edema (DIPE) is considered only after excluding other known causes such as shock, aspiration, and overwhelming pneumonia (1).

The pathophysiological distinction between CCPE and NCPE is crucial. In CCPE, elevated left ventricular pressure causes pulmonary venous congestion, while in NCPE, the alveolar-capillary barrier is directly compromised, typically due to toxins or inflammatory processes. Although both conditions present with similar respiratory symptoms, treatment strategies differ significantly. CCPE is primarily managed with diuretics and inotropes, while NCPE necessitates the cessation of the offending agent and supportive care.

Various drug classes may induce DIPE via distinct pathophysiological mechanisms. For instance, opioids such as fentanyl can lead to central respiratory depression and trigger sympathetic overactivation, thereby increasing pulmonary vascular permeability. Chemotherapeutic agents like cytarabine directly damage the alveolar-capillary membrane through cytotoxic effects, compromising its integrity. Thiazolidinediones, including pioglitazone and rosiglitazone, contribute to fluid retention and increase hydrostatic pressure within the pulmonary vasculature. Meanwhile, calcium channel blockers such as verapamil may induce precapillary vasodilation, resulting in a redistribution of fluid and subsequent pulmonary leakage. These mechanistic insights are critical for understanding how pharmacological agents may precipitate DIPE and underscore the need for vigilance when prescribing these drugs, particularly in vulnerable populations.

Kaplan et al. (2) reported a case of NCPE where DIPE due to Dextran 40 was suspected only after extensive exclusion of other causes, consuming significant time and effort. In complex clinical scenarios, a lack of prior knowledge about drugs with DIPE risk can lead to recurrent or even persistent illness.

Previous studies have suggested that DIPE is often associated with drug overdose, such as heroin, methadone, and propoxyphene (3). Li and Gefter (4) reported three cases of acute pulmonary edema in schizophrenia patients who had overdosed on phenothiazine derivatives. The pulmonary edema resolved within 18 to 40 h, thanks to gastric lavage and supportive care. However, as the use of various drugs has expanded and observation periods have lengthened, DIPE risks have been identified even at standard doses, including drugs like gemcitabine, morphine, and propranolol (1). Haupt et al. (5) observed unexplained fatal pulmonary edema in leukemia patients treated with cytarabine (Ara-C) during the autopsy, which was not found in patients treated with other chemotherapy drugs, such as daunorubicin. This indicates that even drugs with similar therapeutic effects can have different adverse reactions due to their distinct chemical structures. Identifying the DIPE risks of specific drugs and adjusting treatment regimens accordingly is crucial for ensuring effective treatment in high-risk populations.

Post-marketing drug safety monitoring is vital as rare adverse drug events (ADE) escape pre-marketing clinical trial detection. Clinical trials often have limited sample size and follow-up duration, which may only partially capture real-world usage. Previous studies on drugs with DIPE risk are often limited to case reports or clinical observations, which can introduce bias. The Food and Drug Administration (FDA) Adverse Event Reporting System (FAERS), a global, comprehensive, and publicly accessible database, can potentially infer the relationship between drugs and adverse drug reactions (ADRs) (6,7). Given the increasing complexity of clinical presentations and the limitations of anecdotal evidence, a data-driven, large-scale analysis is warranted. This study aimed to fill this gap by systematically evaluating drugs associated with DIPE using the FAERS database, offering a clearer understanding of which drugs present risks in the clinical setting.

## Material and Methods

### Data source

This retrospective pharmacovigilance study was conducted using the FAERS database. FAERS is a self-reporting system where healthcare professionals, consumers, and manufacturers submit adverse event reports to the US FDA to collect post-marketing ADR for drugs and therapeutic biologics. The FAERS database is updated quarterly to identify potential ADRs promptly. In the FAERS database, drug information and different role codes (such as primary suspect, secondary suspect, interacting, and concomitant) are publicly accessible. Since FAERS is publicly available, this study did not require Institutional Review Board approval or informed consent.

FAERS was selected because of its comprehensive nature, offering access to a vast and real-world sample size that is essential for identifying rare ADRs that may not be captured during clinical trials. However, it is important to note that FAERS data may be subject to reporting biases, including underreporting and incomplete information, which should be taken into account when interpreting the results.

This study utilized data from the FAERS database, covering reports from the first quarter of 2004 to the second quarter of 2024. Only reports submitted by healthcare professionals (doctors, medical specialists, pharmacists, and registered nurses) were included. To identify drugs potentially associated with DIPE, we selected “Pulmonary Edema” (MedDRA code: 10037375) as the preferred term (PT) related to DIPE. To eliminate false positives due to misreporting, we excluded reports that included a diagnosis of PE.

### Data analysis

Initially, we focused on drugs identified as primary suspects (PS) in DIPE reports. Only those drugs implicated in at least 10 DIPE cases were considered target drugs. We then conducted a disproportionality analysis based on these target drugs. In this study, we integrated four methods - reporting odds ratio (ROR), proportional reporting ratio (PRR), Bayesian Confidence Propagation Neural Network (BCPNN), and Empirical Bayes Geometric Mean (EBGM) - to identify DIPE signals. A drug was deemed to have a DIPE risk only if all four methods consistently indicated a DIPE signal.

The DIPE risk associated with certain drugs may be subject to population bias. To eliminate potential confounding factors such as age, gender, weight, and report timing, we conducted a multivariate logistic regression analysis. If the report indicated that the individual had taken the target drug, the medication information was coded as 1; otherwise, it was coded as 0. Similarly, if the individual experienced a pulmonary edema (PE) event, the outcome was coded as 1; otherwise, it was coded as 0. For drugs identified with DIPE risk, we extracted the time to DIPE onset from relevant reports and performed statistical analysis. All statistical analyses were conducted using R version 4.3.2. Non-parametric tests were used to compare the differences in ADR onset times between different gender and age groups. Individuals aged ≥65 years were classified as the high-age group, and those aged <65 were classified as the low-age group. A P-value <0.05 was considered statistically significant.

## Results

### Frequency analysis of DIPE

**Figure 1:**
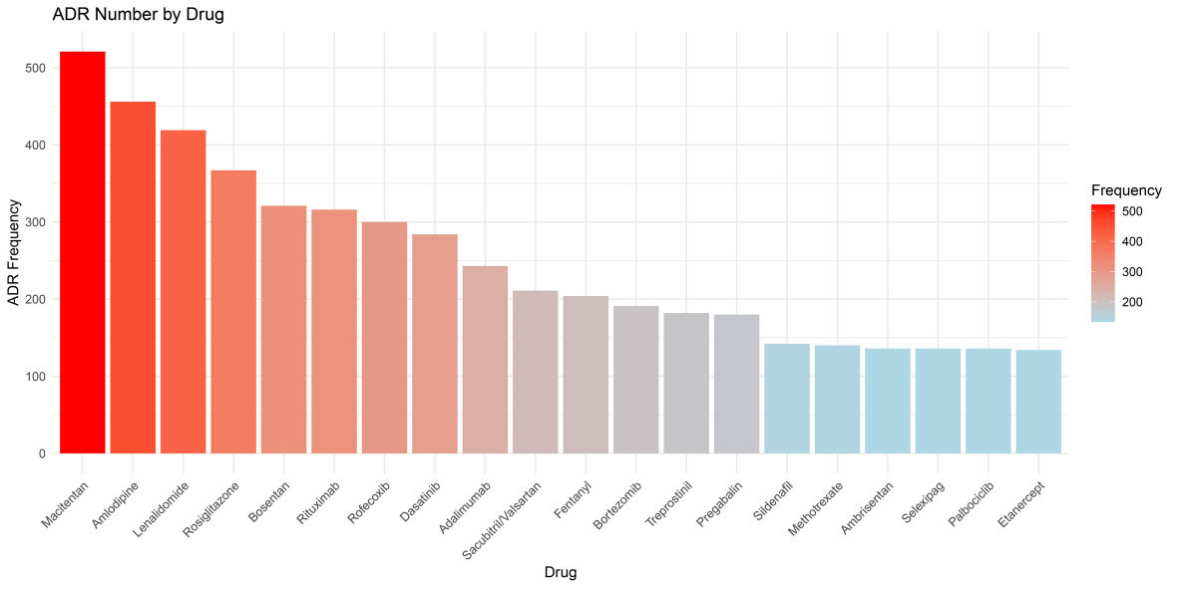
Frequency of drug-induced pulmonary edema (DIPE) adverse drug reactions (ADR). The analysis identified 173 target drugs associated with at least 10 reported cases of DIPE.

By screening drugs identified as PS in at least 10 DIPE reports, we identified 173 target drugs. These drugs were ranked based on the frequency of PE occurrences. The top 20 drugs are macitentan (521), amlodipine (456), lenalidomide (419), rosiglitazone (367), bosentan (321), rituximab (316), rofecoxib (300), dasatinib (284), adalimumab (243), sacubitril/valsartan (211), fentanyl (204), bortezomib (191), treprostinil (182), pregabalin (180), sildenafil (142), methotrexate (140), ambrisentan (136), selexipag (136), palbociclib (136), and etanercept (134). These drugs are designated as target drugs for DIPE. As shown in Figure 1, there are significant differences in the frequency of DIPE induced by different drugs.

### Positive signal identification

**Figure 2:**
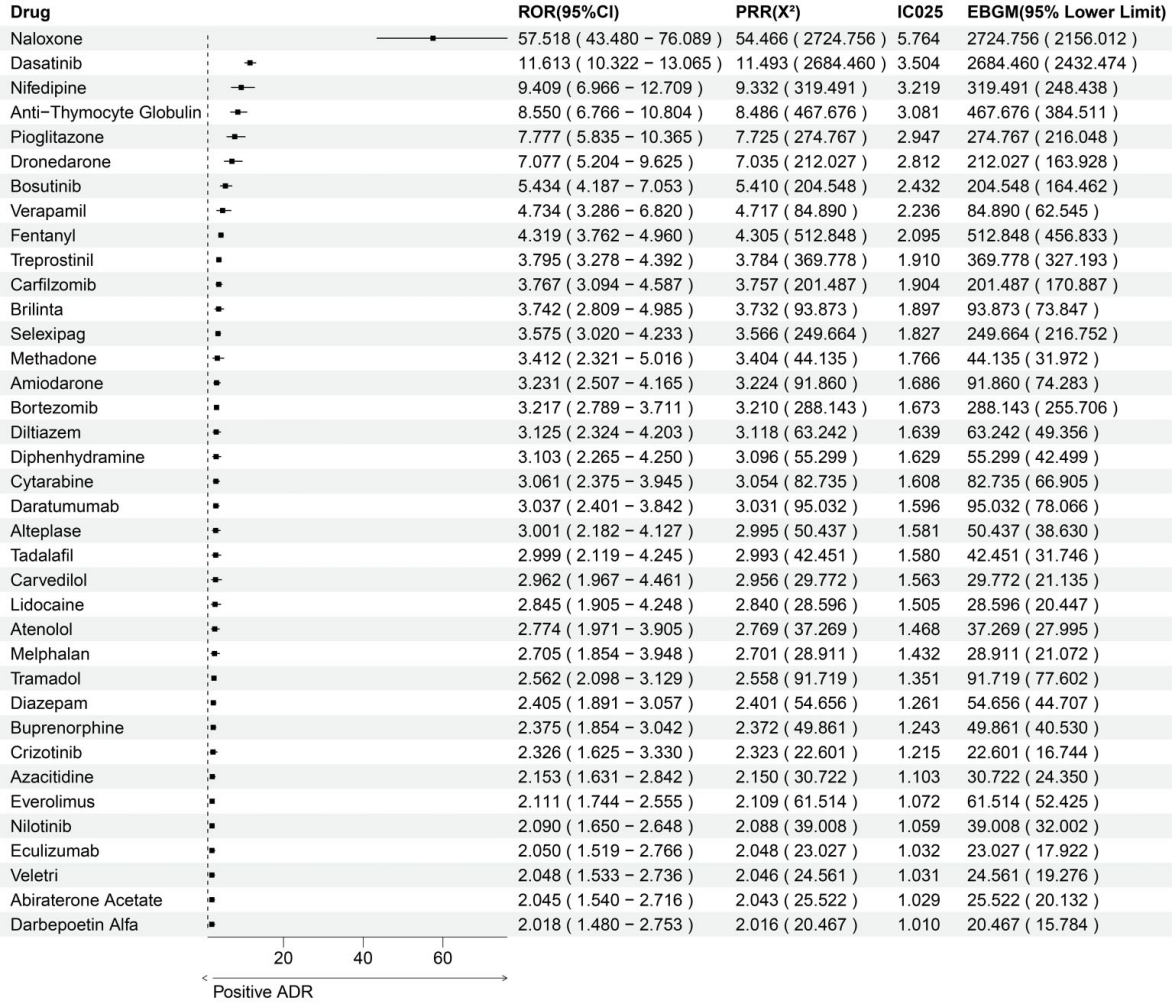
Positive signal identification. After applying four methods (Reporting Odds Ratio (ROR), Proportional Reporting Ratio (PRR), Bayesian Confidence Propagation Neural Network (BCPNN), and Empirical Bayes Geometric Mean (EBGM)), 37 drugs were confirmed to be associated with drug-induced pulmonary edema (DIPE) risk. Notably, naloxone, dasatinib, nifedipine, anti-thymocyte globulin, and pioglitazone exhibited the most significant DIPE signals. ADR: adverse drug reaction.

We employed four methods to identify DIPE signals among drugs comprehensively. Out of 173 candidate drugs, we identified 37 with DIPE risk. The top five drugs, ranked by ROR, are as follows: naloxone, dasatinib, nifedipine, anti-thymocyte globulin, pioglitazone (Figure 2).

### Multivariate logistic regression

**Figure 3:**
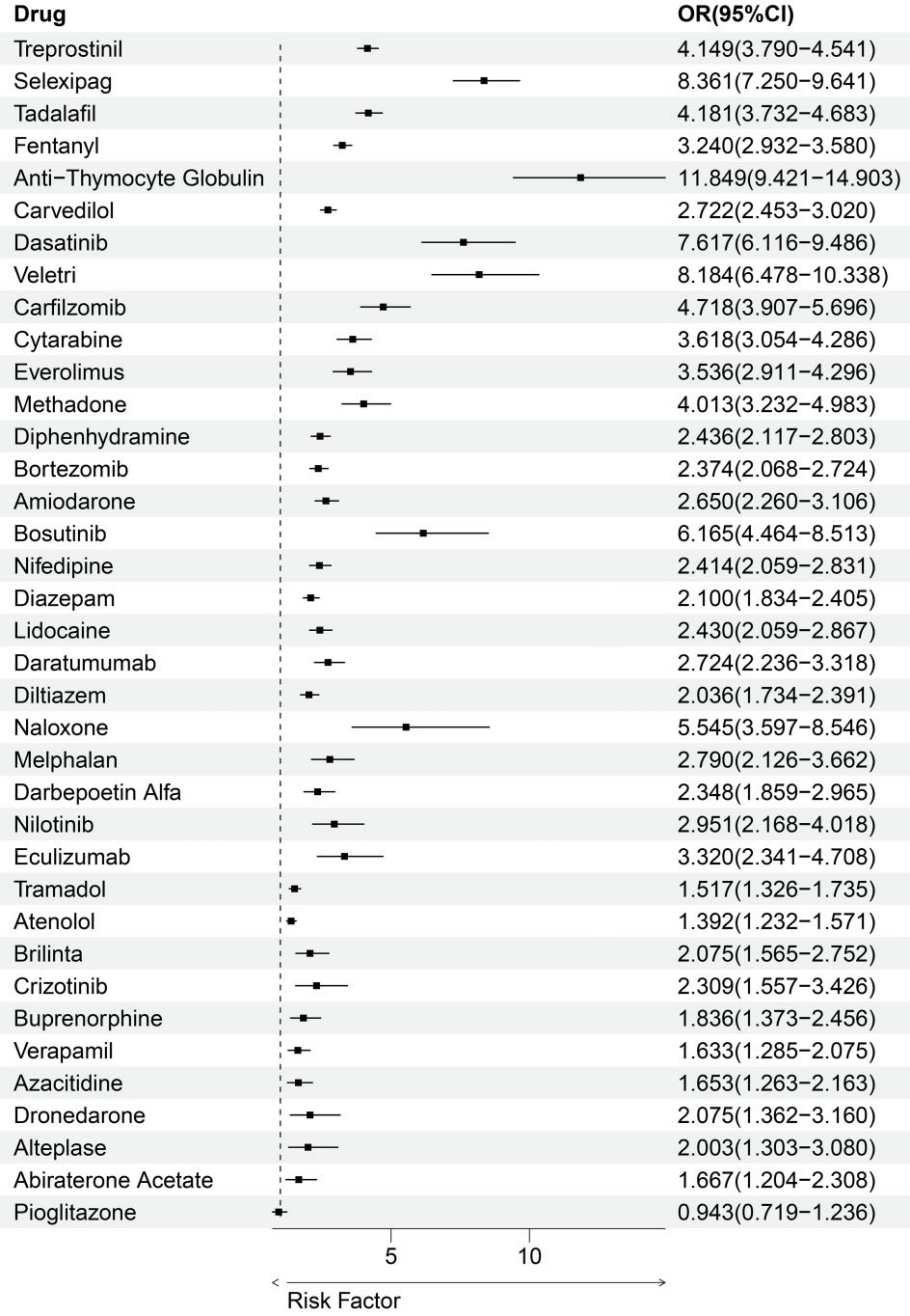
Multivariate logistic regression. Multivariate logistic regression analysis confirmed that 36 out of 37 drugs were significant independent risk factors for drug-induced pulmonary edema (DIPE), indicating a statistically significant association (P<0.05) between these drugs and the risk of DIPE. The only exception was pioglitazone.

To further determine the independence of DIPE risk associated with specific drugs, we conducted multivariate logistic regression analyses on 37 DIPE-risk drugs, using DIPE as a binary outcome variable and adjusting for age, gender, weight, and report time as confounders. The results indicated that, except for Pioglitazone [OR (95%CI): 0.943 (0.719-1.236)], the other 36 drugs were independent risk factors for DIPE (OR>1) with statistical significance (P<0.05) (Figure 3).

### Outcome event analysis

**Figure 4:**
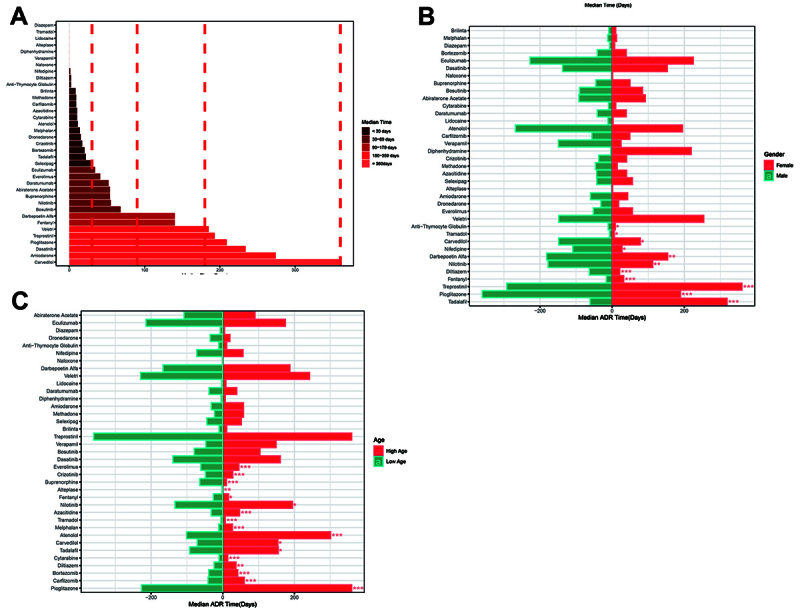
Effect of onset time (**A**), gender (**B**), and age (**C**) on the risk of drug-induced pulmonary edema of drugs. ADR: adverse drug reaction.

Analysis revealed that many DIPE-risk drugs had a median onset time of less than 90 days, which is noteworthy. Furthermore, drugs such as selexipag, tadalafil, bortezomib, crizotinib, dronedarone, melphalan, atenolol, cytarabine, azacitidine, carfilzomib, methadone, brilinta, anti-thymocyte globulin, diltiazem, and nifedipine had a median onset time of less than 30 days (Figure 4A).

We then extracted data with complete age information and found that for the elderly, the mean DIPE onset time was significantly higher after taking pioglitazone, sildenafil, carfilzomib, bortezomib, diltiazem, cytarabine, tadalafil, carvedilol, atenolol, melphalan, tramadol, azacitidine, and nilotinib compared to younger individuals (P<0.05). Conversely, younger individuals had a higher mean DIPE onset time after taking fentanyl, alteplase, buprenorphine, crizotinib, and everolimus compared to the elderly (P<0.05) (Figure 4B).

Additionally, we analyzed data with complete gender information and discovered that males had a significantly higher mean DIPE onset time after taking pioglitazone, diltiazem, nilotinib, darbepoetin alfa, nifedipine, carvedilol, tramadol, and anti-thymocyte globulin compared to females (P<0.05). Conversely, females had a higher mean DIPE onset time after taking tadalafil, treprostinil, and fentanyl compared to males (P<0.05) (Figure 4C).

## Discussion

Drug-induced lung injury is an increasingly common cause of both acute and chronic pulmonary diseases triggered by various cytotoxic and non-cytotoxic drugs. DIPE is one such condition, potentially leading to life-threatening acute hypoxic respiratory failure (8). Early diagnosis is crucial, as discontinuing the offending drug and treating the drug-induced lung injury can significantly reduce morbidity and mortality rates (9). However, the lack of systematic knowledge about drugs that pose a DIPE risk often complicates diagnosis. It is usually considered only after excluding infections, radiation pneumonitis, or recurrence of underlying diseases, which hampers effective disease management.

This study is the first to explore the risk of DIPE based on the FAERS database, employing multiple quality control measures. Given that a higher proportion of reports in the FAERS database come from non-medical personnel, who may lack sufficient medical expertise and thus misreport, we included only reports submitted by healthcare professionals to ensure reliability. Multiple indicators, such as ROR and PRR, were also used for disproportionality analysis. Relying on a single method could increase false positives in drug risk assessment, affecting patient treatment plans. Therefore, we combined various identification methods and identified 37 high-risk drugs for DIPE that warrant clinical attention. Multivariate logistic regression revealed that except for Pioglitazone - whose DIPE risk is influenced by age, gender, or other confounding factors - the other 36 drugs independently contribute to DIPE (OR>1, P<0.05). Consequently, we confidently assert that these 37 drugs can induce PE, with pioglitazone's DIPE effect potentially showing demographic tendencies, meriting further detailed analysis. FAERS, while valuable, is subject to reporting bias. Future studies should experiment with clinical trials to further validate our findings.

Since its FDA approval in the 1970s, naloxone has become widely used as a competitive opioid receptor antagonist to counteract the effects of opioid overdose. However, naloxone can also lead to the development of PE (8). A study reported a 1.2% incidence of PE among 1,456 patients treated with pre-hospital naloxone for suspected opioid overdose (9). If not promptly managed, the severity of naloxone-induced DIPE can range from self-limiting to life-threatening (10). Interestingly, PE can occur even at low doses of naloxone (11,12), and young individuals receiving standard doses are not exempt from this risk (13). These clinical findings are consistent with our own FAERS-based analysis, which confirms that naloxone poses a DIPE risk regardless of age or gender. The mechanism behind naloxone-induced PE is partly attributed to catecholamine-induced vasoconstriction leading to fluid shift (14). However, Elkattawy et al. (8) documented a case where naloxone-induced PE occurred without significant hemodynamic changes, indicating that the precise pathophysiology requires further investigation.

Similarly, our study identified pioglitazone and rosiglitazone as having a DIPE risk. As members of the thiazolidinedione family, both drugs have been linked to PE associated with congestive heart failure (15,16). For instance, Jearath et al. (17) reported a patient who developed congestive heart failure and PE within a year of starting pioglitazone therapy despite having normal left ventricular function. Mudaliar et al. (18) found that the incidence of PE was 15.3% in patients receiving insulin plus pioglitazone and 14.7% in those receiving insulin plus rosiglitazone (compared to 7.0 and 5.4%, respectively, in the insulin-only groups). Besides peripheral edema, studies have also described PE related to thiazolidinedione treatment, with clinical improvement observed only after drug discontinuation. These findings not only support our identification of both pioglitazone and rosiglitazone as DIPE-associated drugs but also underscore the relevance of real-world pharmacovigilance in validating such associations. Interestingly, pioglitazone was the only drug in this study affected by confounding factors, whereas rosiglitazone's risk was independent. This suggests that the safe use of rosiglitazone warrants greater attention.

Verapamil is an effective calcium channel antagonist with various systemic effects, including smooth muscle relaxation, negative chronotropy, and positive inotropy. However, the risk of DIPE from verapamil overdose has been noted (19) even in healthy young individuals (20). Studies suggest that excessive verapamil causes systemic precapillary vasodilation and peripheral edema by affecting vascular smooth muscle. Fentanyl, a phenylpiperidine derivative, can induce PE due to allergic reactions, particularly during surgery (21). Respiratory depression from fentanyl is often reversed with naloxone in clinical settings; however, naloxone may exacerbate fentanyl's DIPE risk. This combined effect has been reported in multiple studies (13,22,23). In line with these observations, we identified strong DIPE signals for both fentanyl and naloxone and, therefore, recommend caution regarding their concurrent use in clinical scenarios.

Among all drug-related deaths, 24.4% involved methadone toxicity (24). PE is a fatal complication of acute methadone overdose, leading to numerous fatalities. An autopsy study conducted in Iran found that 64.1% of 64 cases of methadone toxicity deaths were diagnosed with PE (25). These mortality data provide clinical context for the strong DIPE signal associated with methadone in our study, reinforcing its relevance as a high-risk drug. Dasatinib, a second-generation BCR/Abl tyrosine kinase inhibitor (TKI), has off-target effects that may contribute to adverse reactions such as PE and pleural effusion (26). Similarly, bosutinib has been suspected of causing pulmonary complications, including PE (27). Our results provide supportive real-world evidence for these suspicions, confirming the DIPE association for both agents.

Furthermore, the risks associated with drugs such as cytarabine and amiodarone have been suspected but lack definitive evidence, leaving many drugs “under suspicion” without conclusive “conviction”. Our study helps clarify this uncertainty by providing robust pharmacovigilance data to affirm DIPE risk in these previously ambiguous cases. Previous studies have also implicated drugs like retinoic acid and aspirin; however, due to our strict selection criteria, some lower-risk drugs may yet to be noticed. This does not imply that these drugs are without risk.

Furthermore, this study identified several drugs previously unreported to be associated with DIPE, including clonazepam, dronedarone, treprostinil, and carfilzomib. Analysis of the induction times for DIPE with these drugs revealed age and gender differences, suggesting the need for personalized safety monitoring in clinical use.

Unlike previous FAERS-based studies that have predominantly focused on pulmonary toxicities linked to individual agents or specific drug classes, our study is, to the best of our knowledge, the first to systematically delineate DIPE across a wide range of pharmacological categories. Yang et al. (28) investigated amiodarone-associated pulmonary adverse events, Cortes et al. (29) examined cardiopulmonary toxicities related to BCR-ABL inhibitors, and Jiang et al. (30) analyzed drug-induced interstitial lung disease (DIILD) - a condition that is pathophysiologically distinct from DIPE.

Building on this prior work, our study isolated DIPE as a discrete clinical entity and employed a comprehensive multi-method signal detection framework, incorporating disproportionality analysis, confounder-adjusted logistic regression, and onset time evaluation. This methodology facilitated the identification of both well-established and previously unrecognized DIPE-associated agents, thereby expanding the current understanding of drug-related pulmonary complications.

Leveraging a large-scale, real-world dataset from the FAERS database, our findings revealed that agents such as fentanyl, methadone, and diazepam are associated with a particularly elevated risk of DIPE. These results underscore the need for heightened clinical vigilance, individualized risk assessment, and tailored pharmacotherapy to mitigate adverse outcomes in vulnerable patient populations.

## Conclusion

This study, through comprehensive analysis, systematically and robustly found that drugs such as fentanyl, methadone, and diazepam carry a high risk of DIPE. Despite the insights provided, important knowledge gaps remain, specifically in understanding the mechanisms underlying DIPE onset across diverse populations, and in confirming causality beyond statistical associations. Our study contributes to closing this gap by systematically identifying drug-specific risks in a real-world population, offering a foundation for future mechanistic and prospective validation studies.
